# Examining the impact of the QUARTET USA trial using the translational science benefits model

**DOI:** 10.1017/cts.2024.641

**Published:** 2024-10-31

**Authors:** Guhan Iyer, Danielle Lazar, Abigail S. Baldridge, Jairo Mejia, Clara K. Chow, Namratha R. Kandula, Olutobi A. Sanuade, Linda Rosul, Jody D. Ciolino, Mark D. Huffman

**Affiliations:** 1 Cardiovascular Division and Global Health Center, Washington University in St. Louis., St. Louis. MO, USA; 2 Access Community Health Network, Chicago, IL, USA; 3 Department of Preventive Medicine, Northwestern Feinberg School of Medicine, Chicago, IL, USA; 4 Bluhm Cardiovascular Institute, Northwestern Medicine, Chicago, IL, USA; 5 Westmead Applied Research Center, University of Sydney, Sydney, Australia; 6 Department of Medicine, Division of General Internal Medicine, Northwestern University Feinberg School of Medicine, Chicago, IL, USA; 7 Department of Population Health Sciences, Division of Health System Innovation and Research, Spencer Fox Eccles School of Medicine, University of Utah, Salt Lake City, UT, USA; 8 Northwestern University Data Analysis and Coordinating Center, Feinberg School of Medicine, Northwestern University, Chicago, IL, USA; 9 The George Institute for Global Health, University of New South Wales, Sydney, Australia

**Keywords:** Research impact, randomized trial, hypertension, translational science

## Abstract

Evaluation of benefits beyond quantitative academic outputs is essential in determining translational research value. We used the Translational Science Benefits Model (TSBM) to examine the impact of the QUARTET USA trial using 30 benefits across 4 domains: Clinical, Community, Economic, and Policy. We found that the QUARTET USA trial demonstrated impact in six areas within the Clinical, and Community domains and had potential impact in two additional areas within the Community and Economic domains. Use of the TSBM supports the value of the QUARTET USA trial, which can be used as a template for future cardiovascular trials.

## Introduction

The National Institutes of Health seeks to “improve health, revolutionize science, and serve society,” and the National Center for Advancing Translational Sciences supports translational research so new treatments “reach people faster” [1,2]. These objectives emphasize broader impacts of research that may influence areas such as economy, public policy, or society [3]. Systematically evaluating research impact is especially important in T2 translational research to establish intervention effectiveness and clinical guidelines, which can inform T3 implementation research [4]. Due to methodological challenges, organizational inertia, and resource constraints, academic outputs of T2 studies may not be as robust as those from basic science or T1 studies [5]. However, understanding and evaluating impact beyond academia is essential to supporting the value proposition of T2 research.

The Translational Science Benefits Model (TSBM) is a framework designed to identify and define areas of clinical and translational science that provide benefits to public health and society [6]. The TSBM highlights potential benefits that result from scientific innovations and stresses pathways and mechanisms through which these potential benefits can be realized. The aim of the TSBM is to bridge the gap between research discoveries and practical application by understanding that translational research has value beyond traditional quantitative measures used to assess research value [6]. The TSBM provides a structured approach by guiding researchers, policymakers, and stakeholders in making informed, evidence-based decisions about how to best apply scientific findings in routine settings.

The QUARTET USA trial was a double-blinded, randomized trial conducted at Access Community Health Network (ACCESS), a federally qualified health center network (FQHC) in Chicago, USA. The aim of QUARTET USA was to evaluate whether ultra-low dose quadruple combination therapy lowers blood pressure more effectively compared to standard dose monotherapy in patients with hypertension. The purpose of this report is to evaluate the impact of the QUARTET USA trial, a T2 stage translation trial that focused on evaluating effectiveness and safety of an intervention in a controlled setting, using the TSBM.

## Methods

The methods and results of the QUARTET USA trial have been published [7,8]. Briefly, the trial used a type I hybrid effectiveness-implementation, phase II randomized (1:1), double-blind design to evaluate efficacy and safety of an ultra-low (i.e., quarter standard) dose combination of four blood pressure lowering medications among patients with hypertension who receive care at ACCESS. Participants were recruited and randomized from August 2019 to May 2022. Participants received either a (a) quadpill of candesartan 2 mg, amlodipine 1.25 mg, indapamide 0.625 mg, and bisoprolol 2.5 mg or (b) candesartan 8 mg for 12 weeks. Participants in both groups had open-label amlodipine 5 mg daily added to their regimen at 6 weeks post-randomization if their blood pressure was ≥130/≥80 mm Hg. The primary outcome was between arm difference in systolic blood pressure (SBP) change at 12 weeks. Selected secondary outcomes included between arm difference in diastolic blood pressure (DBP) change at 12 weeks and proportion of add-on amlodipine. Safety and tolerability were also assessed. The primary outcome was assessed in a modified intention to treat population using a linear mixed model with fixed study arm, study visit, and baseline outcome value effects and a random participant effect to account for within-participant correlation. We used a two-sided *p* < 0.05 to define statistical significance without adjustment for multiple testing.

This examination of the translational impact of the QUARTET USA trial was conducted using the Translating for Impact Toolkit Case Study and Impact Profile tools from October 2023 to April 2024. The first and last author (GI, MDH) completed the case study and impact profile and sought review and edits from the other authors. This examination followed a guided process where processes and outcomes from the trial were assessed for impact across the 30 potential benefits and 4 major domains (i.e., Community and Public Health, Clinical, Economic, and Policy) outlined by the TSBM [6]. The distinction between processes and outcomes was also done to differentiate between study actions undertaken in pursuit of the trial versus study outcomes discovered because of the trial. For example, training of research staff and establishing manufacturing partnerships would be considered a process as these are done to enable the research. Results that evaluate hypotheses of primary, secondary, or safety outcomes would be considered outcomes as they are a result of the conducted research. Both processes and outcomes have the potential to generate impact as defined by the TSBM.

## Results

### Summary of QUARTET USA results and process evaluation

Among the 62 randomized participants (n = 32 intervention, *n* = 30 control), mean (SD) age was 52 (11.5) years, 45% were female, 73% self-identified as Hispanic, and 18% self-identified as Black. Baseline mean (SD) SBP was 138.1 (11.2) mm Hg, and baseline mean (SD) DBP was 84.3 (10.5) mm Hg. There was no significant difference in SBP change between the intervention and control arms at 12 weeks (–4.8 mm Hg [95% CI: –10.8, 1.3, *p* = 0.123]). However, there was a significant difference in DBP between the arms (−4.9 mmHg [95% CI: –8.6, –1.3, *p* = 0.009]). Add-on amlodipine rate at 6 weeks was lower in the intervention arm (19% vs 53%, model estimated Odds Ratio = 0.08 [95% CI: 0.02, 0.41], *p* = 0.003). Adverse event rates were similar between study arms [8].

In addition to the primary study results, the QUARTET USA process evaluation found that among the 26 (out of 32, 81%) participants in the intervention arm who completed post-trial surveys, 80% were satisfied with combination therapy, 96% reported that benefits of combination therapy outweighed the risks and that it was convenient to take [9]. Qualitative analysis also showed that both participants and healthcare professionals believed combination therapy reduced perceived pill burden and encouraged medication adherence [9].

### Measuring impact using the translational science benefits model

**Figure 1.**
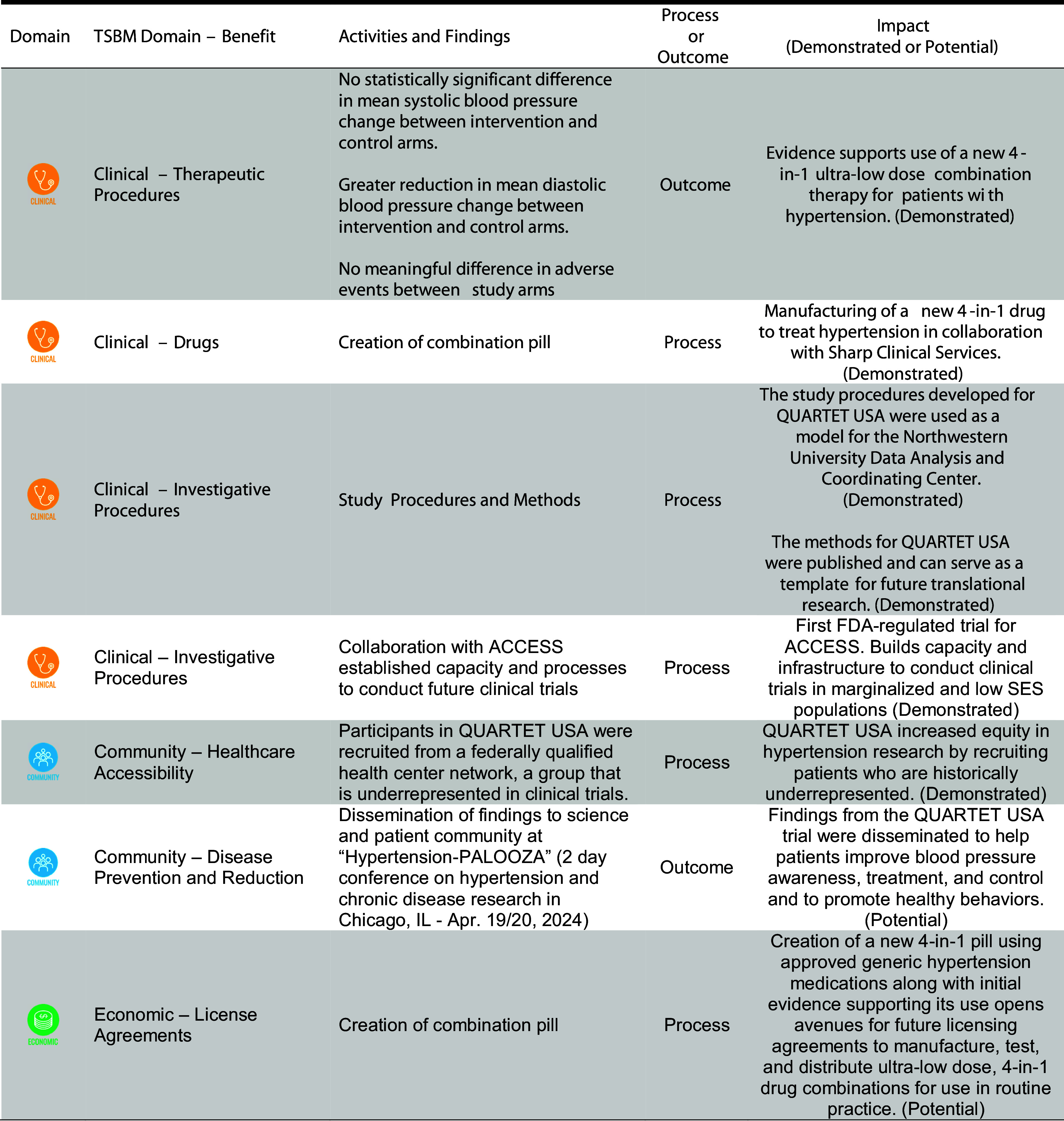
Impact from QUARTET USA trial based on domains outlined by the translational science benefits model (TSBM). ACCESS = access community health network; FDA = food and drug administration.

Figure 1 outlines the benefits from the QUARTET USA trial. There were eight benefits identified in the Clinical and Medical (5 benefits), Community and Public Health (2 benefits), and Economic (1 benefit) domains. There were five benefits in process measures and two benefits in terms of outcomes.

The impacts demonstrated in the Clinical and Medical domain include contributing evidence for the use of ultra-low dose quadruple combination therapy for patients with hypertension (Therapeutic Procedures), the manufacturing of a new 4-in-1 drug combination (Drugs), development of study procedures for data coordination and trial methods (Investigative Procedures), and capacity for Food and Drug Administration regulated trials at ACCESS(Investigative Procedures).

The impacts demonstrated in the Community and Public Health domain include addressing equity in hypertension research by including participants who have been historically underrepresented in research (Involvement in Clinical Trials). The impacts also included dissemination of findings through a science and community dissemination meeting to improve blood pressure awareness, treatment, and control through pharmacotherapy and promote healthy behaviors (Disease Prevention and Reduction [Potential]).

The potential impact in the Economic domain related to evidence created through the QUARTET USA trial that may support future licensing agreements to manufacture, test, and distribute ultra-low dose, 4-in-1 drug combinations for use in routine practice.

## Discussion

Using the TSBM, 8 benefits of impact across 3 domains were identified from the QUARTET USA trial. This evaluation provides the primary, secondary, and safety outcomes reported in the primary study report, as well as the results from the trial’s process evaluation [9].

While the QUARTET USA study failed to reject the null hypothesis for its primary outcome, the direction and magnitude of effect were similar to the related QUARTET trial conducted in Australia, which suggested a –6.9/–5.8 mm Hg average greater SBP/DBP lowering effect at 12 weeks [10]. A study-level meta-analysis of four trials (*n* = 779 participants), including QUARTET and QUARTET USA, supports the blood pressure-lowering effects of ultra-low dose quadruple combination therapy [11]. An individual-level pooled analysis of these three trials is ongoing, which supports the impact wherein the trial contributes to the overall body of evidence related to ultra-low dose quadruple combination therapy.

Most benefits from QUARTET USA were related to the research process, whether through drug manufacturing, trial procedure, conduct and analysis, or inclusion of participants from groups who are historically underrepresented in research. Two benefits are potential and thus should be considered as such. The development of a fixed, ultra-low dose quadruple pill is impactful because it directly addresses low medication adherence and is often able to reduce the need to up-titrate medications [9,12]. These are often mentioned as driving factors for low hypertension control rates. The potential for a licensing agreement to develop this medication is very important because it allows for scalable and sustainable production of the medication independent of grant funding this treatment protocol should be shown to be effective.

The evaluation of research impact is increasing but remains limited. The strengths of the TSBM are its comprehensive yet flexible nature and inclusion of multiple tools to plan, assess, and disseminate impact. On the other hand, this assessment also has potential limitations. First, the TSBM is not the only research impact framework, and it is possible that others such as the Framework to Assess the Impact of Translation health research (FAIT) or the Research Excellence Framework (REF) may provide a better assessment, or at the very least offer a different perspective on assessing impact [13,14]. We selected the TSBM based on our familiarity with the tool and availability of support from Washington University Institute for Clinical and Translational Sciences to use this tool. Second, there is also the possibility that some study impacts do not fit neatly into one of the 30 current benefits identified in the TSBM. For example, the QUARTET USA trial built capacity for FDA-regulated clinical trials at ACCESS through training of research staff as well as support for early-stage investigators. It also allowed ACCESS to build its larger research infrastructure through the use of its electronic health record’s patient portal and SMS texting capacity to reach out to patients who may qualify for studies. These efforts support and enable future participation in clinical trials and healthy equity. Third, the assessment was conducted *post hoc* after trial completion by members of the trial team, which may lead to recall bias or ascertainment bias. However, intimate knowledge of the trial and its process and outcomes is necessary to evaluate impact. An independent assessor may be a more reliable approach than using study team members to carry out these assessments, though no such comparison in evaluating the reliability of impact ascertainment has yet been conducted to our knowledge. Fourth, some benefits may occur in the future, and while we sought to identify those a priori as “potential” benefits, there may be others that we have not anticipated.

In conclusion, we assessed and reported the impact of the QUARTET USA trial using the TSBM, which showed eight benefits across Clinical and Medical, Community and Public Health, and Economic domains. Future research is needed to systematically and prospectively evaluate research impact to help disseminate and translate research into routine practice.
